# High frequency of submicroscopic gametocyte carriage after the treatment of uncomplicated malaria with ACTs

**DOI:** 10.1186/1475-2875-13-S1-P15

**Published:** 2014-09-22

**Authors:** Marielle Karine Bouyou-Akotet, DP Mawili-Mboumba, R Nikiema Ndong, E Kendjo, Brigitte Charron, Valérie Lameyre, Maryvonne Kombila

**Affiliations:** 1 Department of Parasitology Mycology, Faculty Medicine, Libreville, Gabon; 2 Sanofi, Access to Medicines, Paris, France

## Background

Studying the parasite reservoir is a tool for monitoring the effectiveness of control strategies. The gametocyte carriage is more common in patients with asexual forms, and the occurrence of recrudescence or reinfection in patients could also be favored and contribute to the maintenance of a large reservoir of parasites. The aim of this study was to determine the prevalence of submicroscopic gametocytaemia in patients treated for uncomplicated malaria.

## Materials and methods

Gametocytes carriage and density were estimated by Pfs25mRNA amplification using QT-NASBA in samples obtained at enrolment and during the follow-up (day 21 to day 42 post-treatment) of children treated with either artesunate-amodiaquine (ASAQ) or artemether-lumefantrine (AL). Data were analyzed according to the study visit, the presence of asexual parasites, the type of treatment and the treatment response.

## Results

Samples from 48 children were analyzed; 23 were treated with ASAQ and 25 with AL. They had 147 visits, all corresponding to treatment failure with either ASAQ or AL. None of the patients had a microscopic gametocytaemia. Overall, the frequency of SMG carriage was 51%, comparable at day 0 between the ASAQ (53%) and the AL (56%) patients (*P* = 0.6). During the post-treatment visits, it was 58% and 44% in the ASAQ and in the AL groups respectively (*P* = 0.4). When paired samples of 23 children were analyzed, the gametocytaemia was positively correlated with the asexual form density the day of treatment failure (rho = 0.4 in the ASAQ group and rho = 0.5 in the AL). Logistic regression analysis showed that recrudescent infection (aOR: 12.9 [1.1-14.9]) were independent risk factors for SMG carriage whereas no association was found with the type of treatment, age and number of episodes.

## Conclusions

The frequency of SMG carriage is high after ACT treatment whatever the combination used. A strong association between the presence of gametocytes and a recurrent infection is also observed.

